# 

**Published:** 1894-12

**Authors:** 


DECEMBER, 1894.

*Vol. XII.* *No.* **46.**

### THE BRISTOL MEDICO-CHIRURGICAL JOURNAL

**A Quarterly Journal of the Medical Sciences for the West of England and South Wales**

*PUBLISHED UNDER THE AUSPICES OF*
*THE BRISTOL MEDICO-CHIRURGICAL SOCIETY.*

"Scire est nescire, nisi id me

Scire alius sciret."

37477
LIBRARY OF CONGRESS
DEC 1 1897
SMITHSONIAN DEPOSIT

*Editor:* R. SHINGLETON SMITH, M.D., B.Sc.

WITH WHOM ARE ASSOCIATED

J. MICHELL CLARKE, M.A., M.D.
F. R. CROSS, M.B. AND W. H. HARSANT.

*Assistant-Editor:* L. M. GRIFFITHS.

BRISTOL: J. W. ARROWSMITH;
LONDON: J. & A. CHURCHILL, 11 NEW BURLINGTON STREET.

*Price One Shilling and Sixpence.*

